# Person-centred leadership in residential care for older people from the perspective of registered nurses: A qualitative study

**DOI:** 10.1016/j.ijnsa.2025.100377

**Published:** 2025-07-07

**Authors:** Marie Jönsson, Anna-Karin Edberg, Malin Sundström, Anneli Orrung Wallin, Annica Backman

**Affiliations:** a Research Platform for Collaboration for Health, Faculty of Health Science, Kristianstad University, Kristianstad, Sweden; b Department of Nursing, Umeå University, Umeå, Sweden

**Keywords:** Interviews, Leadership, Person-centred care, Registered nurses, Residential care

## Abstract

**Introduction:**

Registered nurses’ leadership involves promoting and implementing the recommended model of care, person-centred care, in residential care facilities. Research on registered nurses’ leadership of person-centred care is, however, limited. The aim was to explore person-centred leadership in residential care for older people from the perspective of registered nurses.

**Methods:**

Focus-group and individual interviews were conducted with registered nurses (*n* = 15) working clinically in residential care facilities in seven municipalities in southern and northern Sweden. All interviews were performed digitally. Data were analysed using qualitative content analysis.

**Results:**

Person-centred leadership meant leading through sense-making person-centredness in a complex care environment. This required both skills and abilities in leading person-centred care, while simultaneously managing various challenges in leading person-centred care.

**Conclusions:**

This study explores the skills required for person-centred leadership and the challenges of applying these skills. The results advance our knowledge by conceptualising person-centred leadership among registered nurses in residential care facilities by sense-making person-centredness in a fragmented organisation. The results indicate that registered nurses’ person-centred leadership is defined by their beliefs, abilities, and actions and not always by their position of authority.

**Implications:**

Person-centred leadership has the potential to improve the person-centred care of older people, which is why the challenges encountered in practice need to be addressed. Knowledge of registered nurses’ experiences of person-centred leadership can be used to improve their skills and abilities in leading person-centred care and to appropriately address the organisational challenges encountered in residential care facilities.

**What is already known**

• Previous research shows that leadership is important to implement and maintain person-centred care in residential care facilities
• Nursing leadership is needed at all levels and settings in healthcare systems
• Nursing leadership could affect both staff and the person who receives care

Alt-text: Unlabelled box

**What this paper adds**

• It highlights that person-centred leadership requires certain skills and abilities for leading person-centred care and addressing challenges when leading person-centred care in a fragmented aged care organisation.
• Registered nurses’ competence and skills are fundamental for leading person-centred care in residential care facilities. This result helps clarify the concept of person-centred leadership in this context.
• Person-centred leadership from the perspective of registered nurses and the challenges they experience needs to be addressed by management and stakeholders in residential care facilities.
• Supporting registered nurses’ person-centred leadership has the potential to promote person-centred care for older people.

Alt-text: Unlabelled box

## 1 Introduction

Nursing leadership is needed at all levels and across all care settings to provide effective and relevant health services for persons in need of care and their families (World Health Organisation, 2021). Strong leadership, which is said to be the foundation for advancement in professional areas where registered nurses (RNs) are active, must be prioritised and improved globally (Klopper et al., 2020). RNs are expected to assume the major leadership role in determining and implementing evidence-informed standards of clinical nursing practice, such as person-centred care management, research, and education (World Health Organisation, 2020b). Previous research has shown that leadership is an important facilitator when implementing and maintaining person-centred care (Ebrahimi et al., 2021; Moore et al., 2017). However, little is known about RNs’ views and experiences of person-centred leadership in residential care facilities in the context of care for older people.

Nursing leadership can be seen as an overarching concept. RNs lead nursing care within their profession but can also be formal leaders such as managers. Nursing leadership has been shown to be a significant factor affecting the nursing workforce and nursing environment in terms of: staff members’ job satisfaction, relationship to work and one another, and health and wellbeing; organisational and environmental factors; as well as productivity and effectiveness (Cummings et al., 2018). Nursing leadership has also been shown to be associated with higher patient satisfaction as well as lower patient mortality, medication error, fewer adverse events, and shorter length of stay in hospital (Hult et al., 2023). Nursing leadership thus affects both staff and the people receiving nursing care.

Clinical leadership is a commonly used concept when it comes to RNs’ leadership. A review of clinical leadership in hospital settings and residential care facilities by Stanley and Stanley (2018) showed that attributes associated with clinical leadership were clinical competence and being a good clinical practitioner. It was also important to be supportive, value- and belief-focused, approachable, a motivator of others, a decision-maker, visible in practice, as well as someone who communicates effectively and acts as a role model with a focus on excellence and high-quality care delivery (Stanley and Stanley, 2018). However, only three of the 27 included studies were from residential care facilities, and it is important to remember that RNs’ assignments in municipal care differ from those in hospital care due to different governing legislation, organisation, roles, and responsibilities (SALAR, 2022). In Sweden, challenges regarding leadership among RNs in municipal care have been evident for quite some time. For example, when asked about their leadership roles, many RNs viewed themselves primarily as leaders of small groups, while 28 per cent did not consider themselves leaders at all (Josefsson and Hansson, 2011). In line with this, Lillsjö et al. (2023) revealed that RNs’ leadership in municipal homecare in Sweden faced challenges when it came to coordinating care for residents and managing care staff with low competence. This can be seen in light of Phelan and McCormack (2016), who noted that caring for older people requires a range of knowledge bases to inform practice and ongoing learning from new situations, to be able to lead and adapt appropriately and to mobilise resources for individual situations. However, a systematic review in a hospital context focusing on clinical leadership revealed that interventions to promote RNs’ clinical leadership are complicated, as these interventions need to address cognitive, interpersonal, and intrinsic competencies as well as psychological empowerment, emotional intelligence, and critical reflexivity skills (Guibert-Lacasa and Vázquez‐Calatayud, 2022). This suggests that leading person-centred care in residential care facilities may entail challenges.

To meet varying needs and provide high-quality care to older people, person-centred care has been proposed as the recommended model (ICN, 2022; WHO, 2020a). Central to person-centred care is the relationship between the professionals and the persons cared for and seeing those persons as capable of making decisions and being involved in their own care (McCormack and McCance, 2017). A review by Kogan et al. (2016) describing the current status of research on person-centred care for older people, regarding existing definitions and prominent elements of person-centred care , concluded that person-centred care is increasingly recognised as central to healthcare, which has implications for how nursing care is led and organised. The way the care provision is led is thus essential when implementing person-centred care (Quasdorf and Bartholomeyczik, 2019; Rokstad et al., 2015). Cardiff et al. (2018) also concluded, in their participatory action research study with nurse managers in a hospital context, that person-centred leadership is a complex, dynamic, relational, and contextualised practice that enables self-actualisation, empowerment, and well-being. Furthermore, Lynch et al. (2018) have developed a person-centred situational leadership framework based on an action research study of staff in residential care facilities. The framework notes the importance of capturing attributes of the leader, for example, harmonising actions with the vision relating to the essence of being, balancing concern for compliance with concern for person-centredness, connecting with the other person in the instant, intentionally encouraging the other person to act, and promoting unity through collaboration, appreciation, and trust. This is in line with a systematic review of the implementation of person-centred care interventions by Ebrahimi et al. (2021), suggesting that managing competing values in teams by engagement and having a flexible organisation with situational leadership were crucial for implementing person-centred care interventions for older people in out-of-hospital settings. A literature review by Byrne et al. (2020) operationalising RNs’ definitions of person-centred care found that having power to practice person-centred care was important, meaning that healthcare systems and environments must be conducive to person-centred care . A meta-synthesis of facilitators of person-centred care in dementia care by Brossard-Saxell et al. (2021) showed that an organisational culture in which the leader was involved in and supported education about dementia diseases and appreciated the employees facilitated the delivery of person-centred care . The previously described review by Byrne et al. (2020) also identified barriers to and enablers of person-centred care and found that workplace culture, policy and practice, organisational systems, environmental workload, ward culture, and leadership were important factors. This highlights the importance of the leader for the delivery of person-centred care , which is closely linked and central to RNs’ professional role. However, the literature review revealed that, from the perspective of RNs in residential care facilities, person-centred leadership is lacking.

### 1.1 Aim

This study aimed to describe person-centred leadership in residential care for older people from the perspective of RNs.

## 2 Material and methods

### 2.1 Design

This study had a qualitative descriptive design and is part of a larger project, Person-Centred Care and Leadership in Residential Care (PERLE), with the overall purpose of exploring person-centred care and person-centred leadership in residential care facilities in Sweden. PERLE includes a wide range of methodological approaches, using both qualitative and quantitative methods and analyses (Edberg and Backman, 2025). This interview study responds to one of the qualitative objectives concerning person-centred leadership in the PERLE project and follows a conventional content analysis approach, as described by Hsieh and Shannon (2005). The consolidated criteria for reporting qualitative research (COREQ) (Tong et al., 2007) were used in this study.

### 2.2 Context

In Sweden, residential care facilities are special accommodations for older people who require support and nursing care around the clock. In 2022, about 14 per cent of people 80 or more years old (mean age, 85 years), mostly women, resided in residential care facilities (National Board of Health and Welfare, 2022). Most residents living in residential care facilities have cognitive impairment and/or neuropsychiatric symptoms or frail health and are dependent on others in their activities of daily living (ADL) (Baxter et al., 2022; Björk et al., 2016). The largest staff groups in residential care facilities are licensed practical nurses (59 %), having a three-year specialised education from upper secondary school or equivalent, and nursing assistants (32 %), often having shorter training. RNs account for about 9 per cent of the workforce (Statistics Sweden, 2019) and have a three-year education at the university level. More than one in three employees are born abroad, and in 2017 about 27 per cent of the total staff in municipal care for older people were temporarily employed (SALAR, 2022). The Swedish National Board of Health and Welfare (2017) emphasises the importance of person-centred care in residential care facilities. Despite this national initiative, it remains unclear to what extent this emphasis has reached the staff in residential care facilities. Thus, not all staff have education or training in person-centred care . For RNs, person-centred care has been included in the registered nursing education as a core competence for the past few years.

**Fig. 1:**
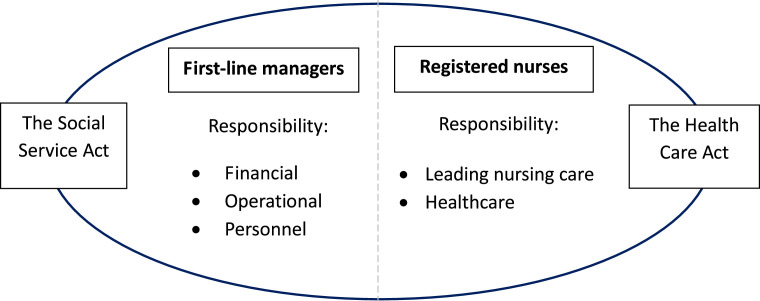
Regulations and responsibilities applicable in residential care in Sweden.

Residential care facility staff are governed by two separate laws. The Social Services Act (SFS 2001:453) outlines the municipality’s responsibility for home care and residential care facilities for older people, and it regulates the work of first-line managers. In contrast, the Health and Medical Services Act (SFS 2017:30) applies to care providers – including municipal, regional, and private organizations – that deliver healthcare services and employ licensed professionals such as RNs. RNs may have their offices at the residential care facility or at an office for all RNs in the area located at a distance from the facility. Still, they are responsible for leading the nursing care in a person-centred way. For an overview of applicable regulations and responsibilities in Swedish residential care facilities, see Fig. 1.

### 2.3 Sampling and procedure

To achieve sufficient variety, data were collected in seven purposively selected municipalities in southern and northern Sweden representing both rural and urban areas. With permission from the heads of Social Services, RNs were invited to participate in the study; they were informed of the purpose at workplace meetings, some of which were virtual, and written information was also provided. We used convenience sampling, and the inclusion criterion was being employed as an RN at a residential care facility in one of the included municipalities. In total, 20 RNs agreed to participate in the study, but five dropped out due to heavy workload, partly related to the Covid-19 pandemic. The final sample comprised 15 RNs who participated in focus groups (*n* = 3) with two participants each or in individual interviews (*n* = 9). The participating RNs had a median age of 43 years, which is slightly lower than the mean age of 45 years for care workers (including both direct care staff and RNs) in Swedish municipalities in 2023 (SALAR, 2023). The sample consisted of 13 women and two men. Each participant had responsibility for a median of 24 residents in their residential care facility. For a detailed description of the sample, see Table 1.

**Table 1:** Characteristics of participants.

Characteristics	*n* = 15
Age, yrs	
Range (md)	28–58 (43)
Gender	
Women/men	13/2
Highest formal education	
Registered nurse	11
Primary healthcare nurse	3
Specialist nurse in dementia care	1
Work experience as registered nurse^1^	
Range, yrs (md)	2–40 (8)
Leadership education^1^	
Yes/no	1/13
Education in person-centred care	
Yes/no	7/8
Type of care facility	
General residential care facility	9
Dementia care facility	4
Short-term accommodation	1
Other	1
Number of residents at the facility^1^	
Range (md)	14–65 (24)

1 Missing data, *n* = 1.

### 2.4 Data collection

Data were collected from November 2021 to December 2022. Before the data collection, two pilot focus-group interviews were conducted, with the first testing the interview guide and the second testing the digital technology. As the participants in the pilot interviews worked in other contexts, these interviews were not included in the study. Participants were offered both focus groups and individual interviews to accommodate logistical issues, partly related to the pandemic. All interviews were conducted digitally by the first author via Zoom or Teams at the participants’ workplaces. The participants were asked to provide demographic information in digital form, in connection with the interviews. The first author moderated all focus-group interviews and either the third, fourth, or fifth author acted as the assistant moderator, posing complementary questions, taking notes, and summarising the discussion (cf. Krueger and Casey, 2015).

First, the purpose of the study was explained, and the participants were asked to describe their professional role. The interviewer explained that her own research interest concerned person-centred leadership. All interviews started with an initial question asking participants to elaborate on their views and experiences of person-centred care , to lead the participants towards the interview topic of person-centred leadership. The interview guide can be seen in Table 2.

**Table 2:** Interview guide.

“What do you think of when you hear person-centred care?”
“Can you tell me what you think of when you hear ‘person-centred leadership?’”
“Can you give examples of what it is or could be?”
“What is your experience of leading person-centred care?”
Probing questions:
“Can you tell me about a situation when you led person-centred care C?”
“Could you elaborate?”
“Could you tell me more?
Assistant moderator summing up and checking comprehension:
“Have I understood you correctly?”

The focus-group interviews lasted 98–140 minutes while the individual interviews lasted 45–114 minutes. The audio-recorded interviews were transcribed verbatim and anonymised. The moderator and assistant moderator had no relationship with the participants before the study.

### 2.5 Data analysis

Data were analysed using conventional qualitative content analysis (Hsieh and Shannon, 2005). Before the analysis, all authors shared their pre-understandings of the research topic. First, all authors read the interviews individually and reflected on their initial impression of the text. The first author then highlighted meaning units describing person-centred leadership using NVivo software (version 1.6.1) (QSR International, 2002). The first and fifth authors performed line-by-line coding of the meaning units, resulting in 16 meaningful clusters. In discussion among all authors, these clusters were grouped into sub-categories and sorted into two main categories. During the analysis process, there was constant iteration between the transcripts, codes, clusters, and possible sub- and main categories. According to Hsieh and Shannon (2005), the analysis can also embrace the identification of relationships between categories. In this study, an overarching theme, uniting the two main categories, is presented.

### 2.6 Rigour

To ensure rigour, we have addressed confirmability, credibility, dependability, and transferability (cf. Shenton, 2004). To address dependability, the study design and data collection have been described, and the same interview guide was used for both the individual and focus-group interviews. Throughout the analysis process, the authors continuously discussed the interview transcripts to reduce the risk of subjectivity and to ensure consistency between transcripts and categories, to achieve confirmability. We used quotations from the interviews to bolster transparency and credibility and to illustrate how the findings captured the participants’ experiences and did not reflect the researchers’ pre-understandings. There were also geographical spread and varied demographic characteristics among the participating RNs, to increase the representativeness and transferability of the findings.

### 2.7 Ethical considerations

The study was approved by the Swedish Ethical Review Authority (EPW 2021–00,413) and performed in line with the Declaration of Helsinki (The World Medical Association, 1964). The participants were informed of the purpose of the study, confidentiality, issues related to research ethics, and the reporting of the findings. There was a risk of the study intruding on the participants’ working time, which would otherwise have been used to support the residents. However, this risk must be balanced against the benefit of their contribution to research, which, from a long-term perspective, will benefit other older people. All focus groups and individual interviews ended with reflections, to capture the participants’ experiences of the interviews. Participants were also invited to contact the first author if concerns emerged after the interview.

## 3 Results

The findings revealed an overall theme, *leading through sense-making person-centredness in a complex care environment*. This required both *skills and abilities in leading person-centred care* and simultaneously managing various *challenges in leading person-centred care* , seen as the two main categories, which had seven and five sub-categories, respectively (see Table 3). Representative and coded quotations are presented in relation to the categories; the code P means person, F focus-group interviews, and I individual interviews, while the number identifies the participant.

**Table 3:** Theme, main categories, and sub-categories exploring person-centred leadership in residential care facilities from the RN’s perspective.

Theme	Categories	Sub-categories
Leading through sense-making person-centredness in a complex care environment	Skills and abilities for leading person-centred care	• Taking personal responsibility for person-centred culture • Living and being person-centred • Adapting supervision to improve understanding • Being present to establish relationships • Supporting reflection to enhance a holistic view • Encouraging staff to consider person-centred nursing interventions • Engaging with other professions and utilising their skills
	Challenges in leading person-centred care	• Leading remotely • Lacking a mandate in a fragmented organisation • Facing difficulties due to varying staff competences • Striving to shift away from a task-oriented culture • Handling lack of agreement

### 3.1 Leading through sense-making person-centredness in a complex care environment

The theme *leading through sense-making person-centredness in a complex care environment* meant working based on person-centred core values and simultaneously mediating them to staff. Sense-making person-centredness as an RN involved taking responsibility and asserting person-centred ways of working, to promote the older person’s participation, wishes, and needs without compromising their care. Sense-making person-centredness as an RN also entailed having an overall view of the plan for the resident and orchestrating it. Despite the multifaceted nature of care practices and the complexities of the organisational structure, RNss were motivated by a profound commitment to leading in a person-centred manner alongside their team members, despite an unclear leadership role. They navigated these challenges with dedication, in spite of the fragmented care organisation, striving to ensure that the needs and perspectives of the older people remained central to their approach.

### 3.2 Skills and abilities for leading person-centred care

This main category highlighted that leading person-centred care required certain characteristics and abilities. For example, the RNs said that taking personal responsibility for the vision constituted an aspiration to work towards a culture of person-centredness, by trying to live and be person-centred themselves when leading person-centred care . Promoting improved understanding and changed perspectives as well as being present were seen as prerequisites for establishing genuine relationships. Making sense of person-centredness also involved supporting reflection among the staff to enhance a holistic view of the residents, encouraging staff to consider and choose person-centred nursing interventions, and engaging other professions and utilising their skills. This main category had seven sub-categories.

#### 3.2.1 Taking personal responsibility for person-centred culture

The RNs described striving to take personal responsibility for person-centred culture in the residential care facility – an important feature of being able to lead person-centred care . They believed that the vision and goal needed to be consistent with one’s own personal beliefs, and be presented and applied to everyone, both the first-line manager and the staff at the residential care facility. The vision, according to the RNs’ descriptions, needed to be consistent with the common goal of the entire facility. They described the vision as involving a culture of person-centredness, being able to present person-centred care as appropriate for everyone’s loved ones:

I have also presented my vision of my residential care facility … I must have a vision of what I want for my facility – my mother should be able to live here. (I5)

#### 3.2.2 Living and being person-centred

The RNs believed that their endeavour to live and be person-centred was necessary for leading person-centred care . Participants said that they always tried to act and be person-centred themselves as part of person-centred leadership, from the minute they entered the doors of the facility. The RNs also said that it was important to have the ability to translate theory into practice, by being able to provide concrete examples of something as abstract as person-centredness (i.e., making sense of it). Cited examples involved having individual conversations with staff in an effort to develop a spark of person-centredness or trying to help the staff understand older people and their needs:

To help the staff to see, to put on new glasses to understand these people that we have to care for, whom we are here for. (I1)

Living and being person-centred, as part of person-centred leadership, could also involve accepting when staff did not have the ability to be fully person-centred, but still to persuade them that the persons cared for were important. It was considered important to be interested in others, empathetic, and able to listen and understand so that the staff felt appreciated.

#### 3.2.3 Adapting supervision to improve understanding

Promoting improved understanding through supervision was described by the RNs as important for leading person-centred care . This included adapting the supervision based on the specific staff members in front of them, for example, students, newly graduated RNs, or temporary workers:

The supervision becomes person-centred because I adapt to their needs, how long they want me to accompany them, and when I can let them go – I adapt. (I2)

The RNs said that being responsive to staff members’ interests and promoting their development was a way to facilitate understanding and adapt to the resident’s wishes and right to self-determination.

#### 3.2.4 Being present to establish relationships

The participants mentioned that they needed to be present to establish relationships, which were described as a prerequisite for leading person-centred care . They said that the purpose of being present was to establish relationships and to have good cooperation with the staff, the older people, and their relatives. By being present, they aimed to provide a sense of security at the facility, for example, staying at the facility as much as possible, and could therefore facilitate understandings of how to improve person-centredness for the residents. For example, they could reach out to the staff every day, having coffee together, sharing schedules, and attending the morning meeting:

It’s a busy job with a lot of administrative tasks, but I try to walk around the facility and say hello, to notice what is happening with/among the residents. (P1, F1)

#### 3.2.5 Supporting reflection to enhance a holistic view

RNs described trying to facilitate staff reflection, to help them understand the care context and apply a holistic view of the residents, as a way of leading person-centred care . RNs could support joint reflection with the staff to improve their understanding of underlying mechanisms in order to improve the residents’ well-being, for example, offering sessions to facilitate reflection and learning about performing personal hygiene in a more inclusive and person-centred way. The participants described how encouraging, supporting, and helping the staff to document the care could enhance reflection on what they were doing and why, making them important aspects of person-centred leadership, deepening their understanding and knowledge of promoting the health and well-being of the older people:

To reflect with the staff on why residents with heart failure need to sit in the heart-bed position, so that they understand the logic. (P2, F1)

#### 3.2.6 Encouraging staff to consider person-centred nursing interventions

An ability considered important when leading person-centred care , according to the RNs, was that of encouraging staff to consider person-centred interventions for the residents. The RNs described how they would return a question to the staff members, who usually had the solutions themselves as they knew the residents best, to facilitate understanding of residents’ needs. For example, when a resident was unwell or experienced anxiety, RNs encouraged staff to take a step back and reconsider the nursing intervention options. RNs also described encouraging staff to choose person-centred nursing measures instead of medical treatments such as sedatives, and noted that a walk, a relaxation exercise, or offering a resident who previously worked night shifts the opportunity to stay up at night were important options:

When the resident sleeps poorly and the staff want to give medicine, we suggest that we observe the resident first. (P1, F2)

I probably do the same as [P1], because once you get a clear picture, you can discuss what to do about this. (P2, F2)

#### 3.2.7 Engaging with other professions and utilising their skills

The RNs said that another important ability was that of engaging other professions and utilising their skills, as a way of leading person-centred care . The participants described how they constantly tried to keep the residents at the centre of the care, involving them, their relatives, and other professionals in decisions. They explained that the RN’s responsibility was to gather all the required information, convey it, and provide feedback about the resident’s care plan – to improve understanding of the residents’ various needs and also involve other professions. One example was getting help from the chief community nurse to improve relatives’ understanding that their loved one had the right to decide for themselves whether or not to use bed rails, or to engage the caretaker at the facility so that the resident could experience meaningful everyday activity by helping put up a shelf:

Involving others in the team who have in-depth knowledge, in order to help the resident in this way – it could be a dementia nurse, staff member, or dietician. (I4)

### 3.3 Challenges in leading person-centred care

This second main category encompassed the challenges in leading person-centred care occurring when the fragmented organisation required RNs to work remotely and with a limited mandate. Other challenges described were dealing with inadequate staff competence or working in a task-oriented culture. RNs also said that they confronted lack of agreement regarding the care of the residents, and desired and strove to overcome this challenge. This main category included five sub-categories.

#### 3.3.1 Leading remotely

According to the RNs, a challenge in leading person-centred care was that they were expected to lead the care remotely, i.e., they were located outside the facility. This was described as particularly challenging when the organisation was such that the staff had to call the coordination nurse or use an e-service to get in touch with the RN:

Working at a distance and the staff having to call coordination nurses can make it difficult when a resident needs quick pain relief. (P1, F2)

Another example cited by the RNs was early in the Covid-19 pandemic when the first-line manager of the residential care facility and the first-line manager of the RNs were working remotely from home. The RNs described feeling left behind, as they had a joint and sometimes a divided responsibility to lead and were therefore dependent on each other. This could make it difficult to convey an understanding of person-centred practices and to address the needs of the older persons.

#### 3.3.2 Lacking a mandate in a fragmented organisation

According to the RNs’ reports, a challenge in leading person-centred care was the lack of a mandate in certain matters and situations. An example described by RNs was when the first-line manager of the residential care facility did not act in certain managerial matters, meaning that the RNs had to take the lead, sometimes in matters outside their area of ​​responsibility. The RNs perceived expectations to act as managers, which could be hard to live up to, as not everyone felt comfortable assuming such a role. Another example was when the first-line manager of the residential care facility made contradictory recommendations:

When the first-line manager, who does not know nursing, makes a recommendation, you have to say no, it is not person-centred or safe for the resident. (P1, F3)

And sometimes he or she listens, sometimes not. (P2, F3)

Due to the fragmented organisation, resulting in staff and RNs participating in separate workplace meetings, miscommunications and misunderstandings could obscure understandings of residents’ needs and of care practices. This could challenge the RNs’ leadership, with the risk that the older people’s needs might not be sufficiently met.

#### 3.3.3 Facing difficulties due to varying staff competences

RNs felt that varying staff competences could be a challenge when leading person-centred care , for example, when staff encountered people with dementia in an inappropriate way or when there were deficiencies in nursing care related to poor staff knowledge. For example, when the staff had limited language skills in Swedish, this could lead to misunderstandings that challenged the person-centred leadership and could also create difficulties when interpreting the needs of residents, negatively affecting person-centred care . When RNs experienced gaps in staff competence, they had to take greater responsibility when leading care to address resident needs:

It’s difficult when there are deficiencies in the nursing care, things that you would expect a licensed practical nurse would have knowledge of. (I8)

#### 3.3.4 Striving to shift away from a task-oriented culture

According to the RNs’ descriptions, a challenge in leading person-centred care was when the care culture was task-oriented, i.e., prioritising practical tasks ahead of the residents and their needs. They perceived this when, for example, the staff prioritised washing the residents’ clothes instead of focusing on residents who were worried or sad, spending time with those who needed attention, or noticing their concerns:

It is difficult to reach the staff when practical things such as unpacking goods comes first. (I9)

The RNs said that they found it challenging to lead person-centred care when there was a lack of awareness of the focus of the care; in that case, the activities and needs of the residents were expected to follow the unit’s routines, instead of the opposite.

#### 3.3.5 Handling lack of agreement

A challenge in leading person-centred care , according to the RNs, was when there was disagreement and lack of understanding regarding a resident’s needs and how best to address them. This could become apparent when, for example, the physician opposed the RN’s assessment of a resident, or relatives did not want their loved one to receive treatment, even though the resident’s health would benefit from it. Other challenges described by the RNs were when the first-line manager of the residential care facility opposed the RN’s assessment that extra staff were needed to monitor a resident, or when staff did not address a resident’s needs and wishes, and the RN’s judgment was ignored:

As an RN, you are expected to lead person-centred care, but it is difficult when not everyone is on board – sometimes even the doctor goes against our assessments. (I3)

## 4 Discussion

The findings revealed an overall theme, *leading through sense-making person-centredness in a complex care environment*, which requires both *skills and abilities in leading person-centred care* and simultaneously managing various *challenges in leading person-centred care .* These results will be further discussed in the following section.

Person-centred leadership in residential care facilities means leading through sense-making person-centredness in a complex care environment. This was evident in the findings as RNs taking responsibility, having an overview of the plans for the residents, and orchestrating these plans, despite their unclear leadership role in a fragmented care organisation. Seen in relation to the person-centred nursing framework of McCormack and McCance (2021), the domain care context describes the significance of supportive organisational systems and the sharing of power and systems that facilitate shared decision-making. However, the fragmented care organisation context in which RNs operate constitutes an obstacle to leading and promoting person-centred care. McCormack and McCance (2021) highlighted the importance of identifying existing systems in the organisation, such as the organisational structure, as they can affect the quality of care. However, previous research on dementia care has shown that support for staff and leaders who championed and modelled person-centred care based on policy documents served as an organisational enabler (Marulappa et al., 2022). Another enabler of person-centred care in dementia care, identified in a systematic review, was staff training in person-centred care (Mohr et al., 2021). Our study also notes the importance of the RN acting as a role model as part of sense-making person-centredness. This was evident in the findings when the RNs highlighted how they worked in a person-centred manner and tried to mediate this to the staff. This is in line with the findings of Moore et al. (2017) regarding barriers to and facilitators of person-centred care , showing that one facilitating factor was leaders serving as role models and “forerunners” by working in a person-centred way. As a person-centred culture should permeate the entire organisation, there is a need for means to increase the organisational support for implementing and maintaining person-centred care and supporting RNs to act as role models.

Person-centred leadership in residential care facilities requires certain skills and abilities. Our findings showed that aspects such as taking personal responsibility for person-centred care and living and being person-centred were important, as they supported the development of a person-centred culture. Seen in relation to the person-centred nursing framework of McCormack and McCance (2021), the domain prerequisites for person-centred care describe RNs’: ability to develop interpersonal skills in terms of non-verbal and verbal communication, professional competence in terms of skills and attitudes, and knowledge of the “self”, i.e., being and becoming person-centred practitioners through clarity of beliefs and values – all of which recall the present findings. Another important ability evident in our findings was that of being present in order to establish relationships, which can be contrasted to the RNs’ narratives of how they mostly provided remote leadership. Andersson et al. (2022) showed that missed nursing care for older people, according to healthcare staff, stemmed from a lack of preparedness for unexpected situations, obstacles in a deficient work environment, unsatisfactory planning in the organisation, and/or shortcomings related to individual staff. However, their results also revealed that healthcare staff wanted to give more to the older persons, but felt restricted by their schedules (Andersson et al., 2022). This is in line with Midje et al.’s (2022) study of staff and RNs, showing that job resources were related to person-centred processes and work engagement, while a Finnish study in residential care facilities showed that person-centred care competence was associated with the person-centred care climate (Pakkonen et al., 2023). Person centred care seems to be interrelated with the caring climate as well as with the work situation of staff, illustrating the importance of RNs having the prerequisites as well as taking responsibility for person-centred culture.

Person-centred leadership in residential care facilities entails several challenges. One challenge seen in the findings was varying staff competences, which meant that RNs had to take greater responsibility to compensate for these shortcomings. This is a well-known challenge in the care of older people in Sweden as well as internationally, with low staffing levels, high staff turnover, and lack of trained staff being realities raising concerns worldwide (OECD, 2020; WHO, 2020c). Previous research has shown that leadership that supports staff in decision-making and treats mistakes as learning opportunities stimulates organisational commitment among staff, creating the potential to increase staff retention and reduce turnover (Gaudenz et al., 2019). Seen in relation to the present study’s findings, person-centred leadership by RNs also has the potential to maintain and safeguard the staffing necessary for person-centred care . This is in line with a study by Backman et al. (2021) showing that residential care facility units with higher levels of person-centred care had a significantly higher proportion of licensed practical nurses than did units with lower levels of person-centred care . This finding has been supported by Kong et al.’s (2022) study, in which staff reported that insufficient education and staffing were obstacles to implementing person-centred care in residential care facilities. This means that leading person-centred care requires that certain educational standards should be maintained and developed and also requires conducive organisational conditions in terms of sufficient staffing levels and an appropriate skill mix. Other challenges described in our study arose when RNs lacked a mandate in a fragmented organisation and when there was disagreement regarding, for example, the need for extra staff. An integrative review by Rajamohan et al. (2019) concluded that person-centred care as a philosophy needs to be understood by all management and staff to be translated into practice. It was stated that it is crucial to develop effective and empowered teams, supported by management, to achieve this. This implies that RNs need to be supported and mandated by management to lead person-centred care based on uniform person-centred care principles upheld throughout the organisation. This assumption was supported by Stewart et al. (2023), who concluded that organisational support could influence work outcomes, including quality of the care for the older people as well as job performance. In addition, Sköldunger et al. (2020) showed that a higher level of person-centred care was related to a lower level of perceived job strain among staff in residential care facilities, with additional implications for promoting person-centred leadership. Despite several challenges encountered in residential care facilities, RNs have an important role in maintaining and increasing competence by overcoming existing obstacles in an attempt to retain and safeguard existing staff. Another challenge to the provision of person-centred leadership in residential care facilities seen in our findings was the need to shift away from a task-oriented culture. This is supported by the results of Guney et al. (2021) showing that a task-oriented organisation and culture hinders the performance of person-centred care . Our findings also highlighted the importance of RNs’ ability to support reflection and foster a holistic view. Structured and ongoing staff discussion and reflection are certainly central to counteracting a task-oriented culture and supporting person-centredness, which in turn can mitigate several of the challenges in leading person-centred care . Although the Swedish organisation of residential care facilities, having two responsible authorities and being governed by two different laws, may limit the transferability of the results, it is still reasonable to believe that the challenges RNs face in terms of the leadership of person-centred care may be universally applicable. Their unclear leadership role, with limited mandate as well as systemic and organisational challenges, made it difficult for RNs to lead the person-centred care in this study. This result is supported by a systematic review finding that RNs across the globe face similar challenges (Compton et al., 2023). Compton et al. (2023) suggested that RNs working in long-term care, equivalent to residential care facilities, are expected to assume leadership roles from the outset but need adequate support and preparation to succeed in these positions.

## 5 Strengths and limitations

Person-centred leadership in residential care facilities is not well defined, and a strength of this study is that it advances our understanding of this concept. The data were gathered from focus-group and individual interviews adapted to accommodate the Covid-19 pandemic. The individual interviews gave the participants the opportunity to describe their experiences in depth, whereas the focus-group interviews could yield new insights and enriched discussions. This combination could be seen as both a strength and a potential limitation. As data were collected in Swedish municipal care, this specific context must be considered when transferring the results to other contexts or settings, including the role that RNs have in Swedish residential care facilities. To gain a more comprehensive understanding of person-centred leadership in residential care facilities, other perspectives, such as those of staff, other professions, and relatives, are also needed.

## 6 Conclusions

This study is novel as it demonstrates the significance and content of RNs’ person-centred leadership in residential care facilities in Sweden and contributes to the conceptualisation of person-centred leadership. A person-centred culture needs to permeate the entire caring organisation, calling for organisational support of the implementation of person-centred care in which RNs can act as role models. Structured and ongoing staff discussion and reflection are central to counteracting a task-oriented culture and supporting person-centredness, which in turn can mitigate several of the challenges in leading person-centred care . The study illustrates the importance of RNs having the prerequisites for person-centred culture as well as taking responsibility for it.

## 8 Implications

The implications for practice and policy are that person-centred leadership exercised by RNs has the potential to improve the person-centred care of older people, which is why the challenges encountered in practice need to be addressed by management and stakeholders in residential care facilities. Thus, supporting RNs’ person-centred leadership seems essential. To support workforce sustainability, the implications for education are that person-centred leadership should be a part of future nursing curricula, including for postgraduate courses as well as continuing education for clinical practitioners. The present findings are exploratory and descriptive and therefore have implications for research as they can serve as a basis for the development of interventions.

## Funding statement

This study was financially supported by the Swedish Dementia Foundation and the Research Platform for Collaboration for Health, Kristianstad University.

## Ethics approval statement

The study was approved by the Swedish Ethical Review Authority (No. 2021–00,413) before we commenced data collection. All participants were given both oral and written information about the study and all participants gave their written informed consent.

## Author details

All authors are RNs and women. All four co-authors have a PhD in nursing and conduct research in the field of care for older people, while the first author is a PhD student. The group has extensive experience of conducting qualitative research.

## CRediT authorship contribution statement

**Marie Jönsson:** Investigation, Writing – original draft, Formal analysis. **Anna-Karin Edberg:** Writing – review & editing, Methodology, Conceptualization, Validation, Funding acquisition. **Malin Sundström:** Writing – review & editing, Investigation, Methodology, Conceptualization. **Anneli Orrung Wallin:** Writing – review & editing, Investigation, Methodology, Conceptualization. **Annica Backman:** Supervision, Investigation, Formal analysis, Writing – review & editing, Methodology, Funding acquisition, Conceptualization.

## Declaration of competing interest

The authors have declared no conflict of interest.
